# Mechanomics: an emerging field between biology and biomechanics

**DOI:** 10.1007/s13238-014-0057-9

**Published:** 2014-04-23

**Authors:** Jiawen Wang, Dongyuan Lü, Debin Mao, Mian Long

**Affiliations:** Center for Biomechanics and Bioengineering and Key Laboratory of Microgravity, Institute of Mechanics, Chinese Academy of Sciences, Beijing, 100190 China

**Keywords:** mechanomics, mechanobiology, proteomics, transcriptomics

## Abstract

Cells sense various *in vivo* mechanical stimuli, which initiate downstream signaling to mechanical forces. While a body of evidences is presented on the impact of limited mechanical regulators in past decades, the mechanisms how biomechanical responses globally affect cell function need to be addressed. Complexity and diversity of *in vivo* mechanical clues present distinct patterns of shear flow, tensile stretch, or mechanical compression with various parametric combination of its magnitude, duration, or frequency. Thus, it is required to understand, from the viewpoint of mechanobiology, what mechanical features of cells are, why mechanical properties are different among distinct cell types, and how forces are transduced to downstream biochemical signals. Meanwhile, those *in vitro* isolated mechanical stimuli are usually coupled together *in vivo*, suggesting that the different factors that are in effect individually could be canceled out or orchestrated with each other. Evidently, *omics* analysis, a powerful tool in the field of system biology, is advantageous to combine with mechanobiology and then to map the full-set of mechanically sensitive proteins and transcripts encoded by its genome. This new emerging field, namely *mechanomics*, makes it possible to elucidate the global responses under systematically-varied mechanical stimuli. This review discusses the current advances in the related fields of *mechanomics* and elaborates how cells sense external forces and activate the biological responses.

## Introduction

Mechanical stimuli are crucial to many biological processes at organ, tissue, cell, and molecule levels. Shear flow, tensile stretch, and mechanical compression are most typical *in vivo* mechanical stimuli, which are in action alone or synergistically with other mechanical and even biochemical factors (Wang, 2006; Cohen and Chen, 2008). For example, endothelial cells are subjected to blood shear flow and orientated towards the flow direction (Silkworth and Stehbens, 1975), extracellular matrices (ECMs) are stretched to mediate the outside-in signaling (Wright et al., 1997), and cartilage tissue is compressed to initiate interstitial fluid pressurization (Soltz and Ateshian, 2000). A body of cues is known about how these mechanical stimuli affect cell morphology, proliferation, and differentiation. Evidently, it is difficult to elucidate what really happen at cellular and molecular levels if only a single type of mechanical stimuli or one set of mechanical parameters at a given stimulus is used.

Homeostatic imbalances are main driving forces to initiate physiological changes, in which various stimuli are monitored closely by receptors and sensors at different sites. Most of mechanoreceptors often react to shear flow, tensile stretch, or mechanical compression. When multiple mechanical stimuli or parameters are pooled together, part(s) of those molecular events presented in individual stimuli tests might be reserved constantly, fostered cooperatively, or canceled out each other, since the cross-talks existing in the mechanically sensitive genes and proteins would exert null, synergistic, or opposite effects. Thus, global mapping of activated genes and proteins that are responsible to the specific mechanical stimuli is required to conduct from the viewpoint of omics. In this review, cellular and molecular responses to mechanical stimuli were discussed, and the new conceptual terminology of mechanomics referred to transcriptomics and proteomics combined with systematically-varied mechanical stimuli was proposed.

## Mechanobiology and Transcriptomics/Proteomics

**Figure 1:**
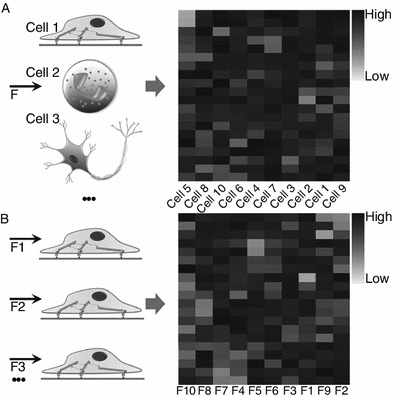
**Schematic of cellular responses to mechanical stimuli in one-to-more (A) or more-to-one pattern (B)**. Each row of the heatmap represents different genes, of which the abundance is indicated by top-right color key, and each column of the heatmap denotes distinct cells (A) and different mechanical forces (B)

Mechanobiology is an interdisciplinary field at the interface of mechanics and biology that emerges over last two decades (Wang et al., 2008). It focuses on elucidating the mechanisms how external forces or changes in cell or tissue mechanical environment contribute to development, physiology, and disease of an organism. A major challenge in this field is to elucidate the molecular mechanisms of mechanotransduction, by which cells sense and respond to biomechanical signals and convert them into biochemical signals (Katsumi et al., 2004; Long et al., 2011; Dado et al., 2012). Numerous mechanoreceptors, such as ECM molecules, transmembrane proteins, cytoskeleton, nuclei, and lipid bilayer, are mechanically sensitive and then initiate inside-out or outside-in mechanotransduction for cells (Janmey and McCulloch, 2007). A single type of mechanical stimuli *in vitro* often provokes cell’s sensing and responding to external forces *via* multiple molecular events (Fig. 1A). By contrast, multiple types of mechanical stimuli are present around a cell or cells *in vivo* and act on the cell(s) synergistically (Fig. 1B). It is also noticed that the physiological stimuli are usually dynamic rather than static, which highly depends on the magnitude, frequency, and duration of mechanical forces.

On the other hand, omics is the most striking tool to globally map the cellular and molecular responses to various biochemical and biomechanical stimuli. It aims, from the viewpoint of system biology, at the characterization and quantification of pooled biological molecules that transmit external signals to alter the structure, function, and dynamics of an organism or organisms. Integration of existing approaches in biomechanics and high-throughput transcriptomics/proteomics enables us to profile cell phenotype under complicated mechanical stimuli and to elucidate the mechanisms in cell type-specific mechanobiology (Davies et al., 2005). Current efforts mainly focus on understanding the transcriptomics/proteomics on the pattern or parametric dependence of one specific type or on the combined types in respective pattern or parameter sets. Here we discuss the combination of these two fields along typical mechanical clues where the detailed parameters are summarized in Table 1 for clarity.

**Table 1:** Summaries of mechanotransduction under typical mechanical stimuli

Stimuli	Types	Pattern	Parameters	Acting cells/tissues	Related molecules	References
Shear flow	Isolated	Laminar/steady	10–20 dyne/cm^2^, 3–24 h	ECs	Integrins, Flk-1, GPCRs, PECAM-1	Li et al. (2005)
			12 dyne/cm^2^, 24 h	ECs	Tie2, Flk-1, MMP1	Chen et al. (2001)
			5–15 dyne/cm^2^, 24 h	ECs	PDGF-BB, TGF-β1, Lamin A	Qi et al. (2011)
			15 dyne/cm^2^, 0–6 h	ECs	PKA, PECAM-1, VEGFR2	Wang et al. (2009)
			12 dyne/cm^2^, 4–24 h	ECs, SMCs	ICAM-1	Heydarkhan-Hagvall et al. (2006)
			3 dyne/cm^2^, 6 h	MSCs	Annexin, GAPDH	Yi et al. (2010)
			12–36 dyne/cm^2^, 40 min	Myeloma ARH-77 cells	Actin	Porat et al. (2011)
		Oscillatory	10 dyne/cm^2^, 1 Hz, 1 h	MSCs	Runx2, Sox9, PPARγ	Arnsdorf et al. (2009)
			12 dyne/cm^2^, 1 Hz, 1 h	Osteoblasts	PGE2	Malone et al. (2007)
			5–50 dyne/cm^2^, 0.5–2 Hz, 1–4 h	Osteocytes	COX-2, RANKL, OPG	Li et al. (2012)
		Pulsating	0.4–1.0 dyne/cm^2^, 5 Hz, 1 h	Osteocytes	NO	Tan et al. (2007)
	Combined	Turbulent w/laminar		ECs	DNA synthesis	Davies et al. (1986)
				ECs	VCAM-1, HSP70	Brooks et al. (2002)
		w/equibiaxial stretch		Fibroblasts	Fibronectin	Steward et al. (2011)
		w/cyclic compression		MSCs	Glycosaminoglycan, Col-II	Schatti et al. (2011)
Tensile stretch	Isolated	Uniaxial	20%, 2–6 days	Fibroblasts	EGF	Lü et al. (2013)
			2%–3%, 0.1 Hz, 48 h	Cartilage	IGF-I	Bonassar et al. (2001)
			10%, 0.1 Hz, 1 h	Tenocytes	Col-I/II, MMP-14, Wnt5A	Jiang et al. (2011)
			3%–9%, 0.5 Hz, 3 days	MSCs	Runx2	Shi et al. (2011)
			5%, 1 Hz, 24 h	MSCs	BGH3, CNN3	Kurpinski et al. (2009)
		Intermittent	3%–5%, 0.5 Hz, 2 h/day, 28 days	MSCs	Sialoprotein-2, Osteocalcin, Osterix	Ward et al. (2007)
		Biaxial	20%, 1 Hz, 24–48 h	SMCs	MAPK	Richard et al. (2007)
			0–10%, 0.3 Hz, 1 h	SMCs	Cyr61	Tamura et al. (2001)
			15%, 0.3 Hz, 1–24 h	SMCs	Cyr61, VEGF-A, MMP-1	Yang et al. (2008)
			2%, 0.2 Hz, 7 days	ESCs	Runx2, Sox9	Li et al. (2013a)
			8%, 1 Hz, 2 h	Chondrocytes	eEF1D, ERK	Piltti et al. (2008)
		Equiaxial	6%–14%, 9 days	ACL	Pro-MMP-2	Zhou et al. (2005)
			3%–12%, 1 Hz, 2–24 h	Astrocytes	TGF-β, p53, ANXA4	Rogers et al. (2012)
	Combined	Uniaxial w/equiaxial		SMCs	Rac	Katsumi et al. (2002)
		w/hydrostatic pressure		Chondrosarcoma cells	PDGF-B, Integrin, MMP-3, TGF-α	Karjalainen et al. (2003)
Mechanical compression	Isolated	Static	30 MPa, 6 h	Chondrocytes	HSP 70	Kaarniranta et al. (2003)
			30 MPa, 0–24 h	Chondrocytes	DAP3, PTZ-17, H-NUC, HSP 70	Sironen et al. (2000)
			30 MPa, 6 h	Chondrosarcoma cells	Osteonectin, Fibronectin, c-jun	Sironen et al. (2002)
		Cyclic	5–30 MPa, 0–0.5 Hz, 20 h	Chondrocytes	Proteoglycan	Lammi et al. (1994)
			1.67 MPa, 1 Hz, 24 h	Osteoblasts/Osteocytes	MMP-3, MMP-13, 14-3-3ε	Priam et al. (2013)
			20 N, 2 Hz	Bones	Raf1, PDCD8	Li et al. (2011)
		Intermittent	8 kPa, 0.33 Hz, 4 h/day, 7 days	MPCs	Proteoglycan, Collagen	Angele et al. (2004)
Microgravity stimulation		Head-down-tilt bed test	30 min/day, 5 days	Male volunteers	fMLP, TNF-α, CD62L	Feuerecker et al. (2013)
		Reorientation	5 days	Osteoblasts	Actin, Vimentin	Li et al. (2010)
		Reorientation	135°, 0–60 min	Arabidopsis root apex	HSP70, KNAT1, EF1-a	Kimbrough et al. (2004)
		Reorientation	90°, 30 min	Arabidopsis	GAPDH, P450	Moseyko et al. (2002)
		Rotation	12 rpm, 24–72 h	Lymphoblastoid cells	miR-150, miR-34a, EGR2, ETS1	Mangala et al. (2011)
		Diamagnetic levitation	5 × 10^−2^ g, 1–22 days	Drosophila	P450-6a8, HSP70, Peroxiredoxin 2540	Herranz et al. (2012)
Topography/stiffness/size		Pillar, Groove, PA gel	5 days	MSCs	Runx2, β3-tubulin, Actin, Vimentin	Li et al. (2013c)
		Pits, Grooves	14–21 days	MSCs	MAPK, FGF, PDGF	Dalby et al. (2008)
						Biggs et al. (2008)
		Hydroxyapatite	2–4 days	Osteoblasts	Myosin-9, Filamin-B, Vimentin, Cofilin-1	Xu et al. (2008)
		Grooves	24 h	Fibroblasts	EEF1D, IDH3, UCHL1, PCNA	McNamara et al. (2012)
		Magnetic bead	0.5 mT, 1 Hz, 6 h	Myoblasts	Galectin-1, Annexin III, RhoGDI	Grossi et al. (2011)

ACL, anterior cruciate ligament; EC, endothelial cell; MPC, mesenchymal progenitor cell; MSC, mesenchymal stem cell; SMC, smooth muscle cell

### Mechanical modeling of a cell

Cells sense their mechanical environment, which, in turn, regulates their functions. Various theoretical models are proposed to understand mechanical regulation of cell functions, which provide differential mathematical solutions. At subcellular level, most models are attempting to predict the force or stress distribution of cytoskeletal network and mimic the remodeling of mechanically sensitive cytoskeletal proteins (Ingber, 1997; Li, 2008; Geiger et al., 2009). At cellular level, a cell is usually modeled as an encapsulated lipid membrane containing stress-supported structures to support its deformation, adhesion, and spreading (Evans and Yeung, 1989; Stamenovic and Ingber, 2002; Murrell et al., 2011). At tissue level, mathematically models are developed to predict the dynamics of tumor growth (Chaplain et al., 2006) and osteogenic differentiation (Carter et al., 1998) under mechanical stimuli. These models also help to elucidate the mechanisms how cells resist shape distortion and maintain their structural stability and how they convert mechanical signals into biochemical responses. Regardless of their advantages in theoretical prediction, these models are still required to compare with the relevant experimental measurements.

### Single type of mechanical stimuli

Current works on mechanobiology and mechanotransduction are mainly focused on understanding how the cells sense and respond to a single type of mechanical stimuli and what the functional molecules are only on the basis of a few protein effectors.

#### Shear flow

Cellular responses to shear flow that mimics the physiological blood or interstitial flow are extensively investigated (Liang et al., 2008; Cui et al., 2011; Fu et al., 2011a; Kang et al., 2012). Exposure of live cells to shear flow induces remarkable changes in cell morphology, adhesion, and spreading (Yang et al., 2009; Zhan et al., 2012). For example, endothelial cells (ECs) display adaptive remodeling in response to shear stress, under which surface mechanosensors of integrins, Flk-1, ion channels, GPCRs, and PECAM-1 are involved in sensing shear stress (Li et al., 2005; Barakat and Lieu, 2003). Steady flow enforces myeloma cells to form actin-rich but microtubule-lacking protrusions in a stress-dependent pattern (Porat et al., 2011). Osteocytes subjected to pulsating flow appear to be more responsive than osteoblasts or periosteal fibroblast osteocytes in inhibiting osteoclast formation and resorption *via* NO-dependent pathways (Tan et al., 2007). Shear-induced transportation of circulating tumor cells to the target site is the prerequisite for organ-specific metastasis under blood flow (Wirtz et al., 2011) and tumor cell invasion is usually promoted by interstitial fluid affecting their interactions with stromal cells (Shieh et al., 2011).

Transcriptomic analysis is growing up in cell mechanobiology studies under fluid flow in the past decades. A specific pattern or parameter setting of shear flow initiates the expression of multiple genes in various biological processes. For instance, gene expression profile of ECs subjected to laminar or steady flow proposes the significant modulation of the genes involved in cell proliferation, ECM/cytoskeleton remodeling, angiogenesis, or in inflammatory cytokines, stress response proteins, and signaling molecules (Chen et al., 2001). Oscillatory flow up-regulates the transcription factor expression (Runx2, Sox9, and PPARγ) and induces osteogenic differentiation *via* RhoA and its effector protein ROCK II for the fate determination of mesenchymal stem cells (MSCs) (Arnsdorf et al., 2009). Meanwhile, different patterns or parameter settings of shear flow promote the distinct transcriptomic alternations since distinct gene profiles and resultant phenotypes of ECs are observed between low-shear disturbed flow and high-shear laminar flow to identify >100 new genes (Brooks et al., 2002). Moreover, the physiological conditions or biochemical microenvironments that cells reside should be taken into account under blood flow. One example is that the monoculture of ECs up-regulates ICAM-1 expression but the co-culture of ECs and SMCs down-regulates ICAM-1 expression under laminar flow (Heydarkhan-Hagvall et al., 2006). TNF-α-induced gene expression is distinct from that induced by disturbed flow, implying that the downstream effect of disturbed flow is not mediated as same as the signaling pathways that activate NF-κB (Brooks et al., 2002).

While much progresses have been achieved in transcriptomic analysis under shear flow, a few works are also done in proteomic profiling of cells under shear stress. Under steady flow, 10 and 3 proteins are found to be up- and down-regulated for hMSCs, respectively, in which annexin A2 and GAPDH are substantially increased (Yi et al., 2010). Besides, cells can also sense shear stress and convert it into secretary signals, as seen in 43 differential proteins found from low shear-induced rat thoracic aorta in which two secretary molecules of PDGF-BB and TGF-β1 are critical in vascular remodeling (Qi et al., 2011). Again, the combination of biochemical factors with biomechanical stress is critical, since high glucose alone significantly modulates shear-induced mechanosensing complexes and protein phosphorylation pathways of endothelium while both laminar shear and high glucose together enhance HSPs and protein ubiquitination of bovine ECs (Wang et al., 2009).

#### Tensile stretch

Mechanical stretch usually induces significant changes in cellular responses and tissue remodeling at molecular and cellular levels (Tamura et al., 2001; Zhou et al., 2005; Richard et al., 2007). Static stretch induces asymmetric migration of keratinocytes cocultured with fibroblasts in a wound repairing model (Lü et al., 2013). Tendon fibroblasts sense cyclic stretch in the dose- and time-dependent patterns and induce protein productions (Col-I, TGF-β1, COX, PGE2, and LTB4) (Wang, 2006). Equi-biaxial stretch promotes higher pro-MMP-2 production and its active form in anterior cruciate ligament than those in medial collateral ligament fibroblasts but no difference in post-translational modification is observed in between (Zhou et al., 2005). Transient increase of cysteine-rich protein 61 (Cyr61) mRNA is observed for fetal bovine bladder smooth muscle cells (SMCs) subjected to cyclic biaxial stretch, which is correlated with intracellular signaling (PKC, PI3K, and Rho kinase) (Tamura et al., 2001). Moreover, ion channels could serve as candidate mechanosensors, since cyclic stretch is able to trigger the gadolinium-sensitive stretch-activated ion channels, inducing a rapid Ca^2+^ influx, and then play a crucial role in mechanotransduction of fetal rat lung cells (Liu et al., 1994) with specified structural alterations and gating dynamics (Martinac, 2004).

Gene expression is globally mapped under physiologically-mimicking stretch and a number of mammalian and plant genes reacting to mechanical stretch are identified. Different mechanosensitive genes are defined for the metabolism of chondrosarcoma cells exposed to continuous cyclic stretch (Karjalainen et al., 2003) or for the biochemically-induced osteogenesis and bone nodule formation of human ESCs exposed to intermittent cyclic stretch (Li et al., 2013a). In addition to obtaining a candidate list of the differential genes, integrative knowledge of proteins encoded by mechanosensitive genes and of their interactions with putative partners uncovers the programming of genes functionally involved in paracrine signaling of angiogenesis for bladder SMCs subjected to cyclic stretch (Yang et al., 2008). Nowadays, it is also able to profile plant transcriptomes and compare the genes of interest under mechanical stretch. The first expression sequence tags of tension wood are produced from *Populus tremula* × *P. tremuloides* tension wood cDNA library (Sterky et al., 2004) and four different cDNA libraries are then constructed from both tensile and opposite wood of bent poplars to identify the highly expressed genes from analyzing the tags in the libraries (Dejardin et al., 2004).

Differential proteins under mechanical stretch are also profiled, especially for those events undetectable in transcriptomic analysis. Cyclic biaxial stretch initiates multiple phosphorylations associated with the newly-identified mechanosensitive proteins in chondrosarcoma cells (Piltti et al., 2008) and promotes 194 and 177 differential proteins related to ECM production, intracellular signaling, cytoskeletal remodeling, and inflammatory response from tenocytes cultured on polyglycolic acid long fibers, respectively (Jiang et al., 2011). Cyclic equiaxial stretch uncovers a few consistent major proteins (TGF-β1, TNF, CASP3, and p53) to hub at the center of the interacting network with the newly-profiled proteins of interest (BAG5, NO66, and eIF-5A) in activated lamina cribrosa cells (Rogers et al., 2012), recapitulating the importance of MAPK and TGF-β signaling pathways in mechanotransduction. Biomechanical and biochemical regulations are also coupled together in stretch-induced proteins, as seen that the TGF-β1-activated up-regulation of α_3_β_1_ integrin and uniaxial stretch-induced increase of calponin 3 protein are different from the synergistic up-regulation of calponin 1 gene for human MSCs (Kurpinski et al., 2009). In plant, 5 and 12 proteins are specified in the differentiating tissue of tension-induced wood in *Eucalyptus camaldulensisL*. (Baba et al., 2000) and in the tensile wood of *Eucalyptus gunnii* associated with growth strain (Plomion et al., 2003), respectively. Although the available data for plants is much less than those for mammals, such the mechanically-induced proteomic analysis broadens the mechanotransductive extents in plant sciences.

#### Mechanical compression

Cells are able to sense and respond to internal or external compression. Articular cartilage is a hydrated soft tissue for bearing diarthrodial joints and therefore serves as the main target in compression-induced mechanotransduction. Continuous hydrostatic pressure induces stress-associated transcription factors in primary and immortalized chondrocytes, presumably resulting from the stabilization but not the synthesis of HSP70 mRNA (Kaarniranta et al., 2003). Both dynamic compression and insulin-like growth factor I (IGF-I) regulate, *via* distinct activating pathways, the metabolic activity of articular chondrocytes in a way that dynamic compression accelerates the biosynthetic response to IGF-I and increases transport of IGF-I into the articular cartilage matrix (Bonassar et al., 2001). Tumor is another typical target mainly due to its uncontrolled growth in a confined space. Tumor cells often experience mechanical compressive stress that stimulates adhesion and migration by a subset of “leader cells” (Tse et al., 2012) whereas tibial compression inhibits the growth and osteolysis of secondary breast tumors (Lynch et al., 2013). These studies provide cues for the potential signaling pathways in response to mechanical compression by these mechanosensitive effectors.

Differential gene expression under mechanical compression is one of key issues in chondrocyte mechanotransduction. Chondrosarcoma cells are more likely sensitive to continuous pressure with several induced genes than cyclic and static pressure with few gene changes under different compression regimens and parameters (Sironen et al., 2000). Not only the hydrostatic pressure regulates the gene expression but it also manipulates the mRNA stability of chondrosarcoma cells, since such immediate-early genes as c-jun, jun-B, and c-myc become up-regulated but destabilized under pressure treatment (Sironen et al., 2002).

Proteomic studies of mechanical compression are applied to understand how different proteins are orchestrated to respond. Proteomic analysis between normal and fatigue axial compressive loads for ulna yields 42 differential proteins encoded by 21 genes that produce an interaction sub-network for differentially expressed proteins (mainly for Raf1 and PDCD8) (Li et al., 2011). Co-culture of osteoblasts-osteocytes exposed to cyclic compression induces the protein release of MMP-3 and -13, inhibits the mRNA expression of Col-II and aggrecan, and promotes 14-3-3ε as a new soluble mediator between subchondral bone and cartilage in osteoarthritis, implying the interactive communications among different types of bone cells (Priam et al., 2013). Moreover, cyclic compression also induces differential productions of typical proteins (aggrecan, Col-I/II, and proteoglycan) for bone marrow-derived mesenchymal progenitor cells (Angele et al., 2004).

#### Other mechanical stimuli

There are numerous other mechanical stimuli existing in physiology. Combination of both transcriptomic/proteomic analyses and mechanical loading is also important in those mechanical stimuli. Here are two examples.

Gravitational alterations in space are crucial for astronauts’ bone loss and immune suppression under microgravity, which highly depends on the direct and indirect effects of gravitational changes on the relevant cells observed from space missions or ground-based studies (Zayzafoon et al., 2005; Sun et al., 2008; Li et al., 2010; Feuerecker et al., 2013). While gravity-sensitive genes and proteins are occasionally found to be either up-regulated or down-regulated, there is still lack of systematic studies on gene expression and protein production for various types of cells exposed to different microgravity levels (Nichols et al., 2006). The pioneering works of whole-genome analysis on gravitropic stimulation are performed to delineate the transcriptional response mechanisms of plant growth, where *Arabidopsis* seedlings and root apices in response to clinostat (90° reorientation) and transient gravitropic reorientation (135° reorientation) at different time points (2–60 min) reveal the induction of complex gene expression patterns as a consequence of the fast and transient transcriptional networks and the gravity-induced genes (Moseyko et al., 2002; Kimbrough et al., 2004). Later, the expression profile of microRNAs and genes of human lymphoblastoid cells on simulation of microgravity effect is conducted after being exposed to a rotating wall bioreactor, in which miR-150, miR-34a, miR-423-5p, miR-22, miR-141, miR-618, and miR-222 are found to change significantly (Mangala et al., 2011). Diamagnetic levitation simulation verifies the delay in the development of *Drosophila melanogaster* from embryo to adult and the significant changes in the transcriptional profile of immune-, stress-, and temperature-related genes (i.e., HSPs) (Herranz et al., 2012), implying the gravity-dependent organism development.

There is an increasing evidence that cells respond to the topographical substrate with physiologically-mimicking stiffness in morphology, proliferation, and differentiation (Li et al., 2013c), suggesting that mechanical features of substrate also play a role in mechanobiology and alter cell transcriptomics/proteomics. Genetic profile of hMSCs presents the promotion of the topography-induced bone formation on nanoscale pitted surface and raised islands (Dalby et al., 2008) and yields a similar expression of p38 MAPK molecule on two width-varied microgrooves but a differential regulation of increased PDGF and integrin expressions and of enhanced VEGF signaling on either microgroove (Biggs et al., 2008). Quantitative proteomics on different topographies uncovers 21 differential proteins related to cell cytoskeleton, metabolism, signaling, and growth identified for osteoblasts placed on planar and carbon nanotube reinforced hydroxyapatite surface (Xu et al., 2008). Further studies integrate the transcriptomics and proteomics analyses to provide the consistent data for transcripts and proteins of fibroblasts on grooved substrate in regulating chromatin remodeling (e.g., HMGA1) and DNA synthesis (e.g., PCNA) (McNamara et al., 2012). Additionally, ECM/cell stiffness is an intrinsic mechanical stimulus that can also regulate cell phenotype. As compared to those dispersed cells, periosteal cells residing in stiffness-varied regions yield the differential genes and the related proteins in regulating ECMs, suggesting a negative correlation between stiffness and differentiation (Horimizu et al., 2013). Mechanosensor-based targeting of membrane stiffness provokes 13 proteins involved in the differentiation of embryonic muscle cells, including galectin-1, annexin III, RhoGD I, and FAK phosphorylation (Grossi et al., 2011). Evidently, ECM/cell topography and stiffness are associated with transcriptomic/proteomic changes in signaling pathways and cellular functions, as well as in metastatic potential of tumor cells (Pozo et al., 2007; Swaminathan et al., 2011; Huang and Ingber, 2005).

### Combined mechanical stimuli

Physiologically, the different types of mechanical stimuli or the variety of mechanical patterns or parameters on the same mechanical stimulus are usually coupled together to modulate the cellular and molecular events. Multiple genes and protein effectors are mediated to respond to the acting stimuli or parameters.

#### Differential regulation of patterns or parameters on single type of stimuli

Increasing evidences indicate that cells respond to mechanical stimuli in a pattern-dependent manner. For example, laminar shear induces EC alignment along the flow direction without initiating cell proliferation whereas turbulent shear stimulates endothelial DNA synthesis in the absence of cell alignment (Davies et al., 1986). Exposure of fibroblasts to oscillatory flow does not promote the development of F-actin stress fibers while the actin polymerization and actin stress fiber formation are fostered under steady flow (Malone et al., 2007). Meanwhile, equi-biaxial stretch inhibits lamellipodia formation *via* deactivation of Rac signaling whereas uniaxial stretch suppresses lamellipodia along lengthened sides but increases at adjacent ends (Katsumi et al., 2002). Cyclic pressure enhances the aggrecan mRNA expression while static pressure reduces the aggrecan level in primary chondrocytes (Lammi et al., 1994). Moreover, cells respond to mechanical stimuli in a parameter-dependent manner at a given pattern of specific type. Under oscillatory flow, COX-2, RANKL, and OPG mRNA expressions in osteocytes are sensitive to the combination of peak shear stress (0.5, 1, 2, and 5 Pa), oscillatory frequency (0.5, 1, and 2 Hz), and action duration (1, 2, and 4 h) (Li et al., 2012). Continuous tension at 3%–9% strain for 10 days inhibits MSC differentiation while intermittent tension in minutes or hours per day promotes MSC differentiation *via* Runx2 expression and MAPK signaling (Ward et al., 2007; Shi et al., 2011). These data illustrates that cellular responses are highly sensitive to the patterns and/or parameters of mechanical stimuli that are imposed.

#### Combinatory impacts of different types of stimuli

Cells usually undergo *in vivo* distinct types of mechanical loads that are inextricably coupled and are put into effect synergistically. For example, biomechanical tests of torsion-tension of cadaveric femurs reveals the simultaneous, coupled impacts of mechanical torsion and tension (Zdero et al., 2011). Tensile strain amplification from tissue to cellular level in bone is induced by fluid drag forces on bone cells (You et al., 2001), implying the cross-talk between tensile stretch and shear flow. When an equi-biaxial tension and a steady flow are applied separately to fibroblasts in the presence or absence of RhoA inhibitor, the mechanical stimuli regulate fibronectin reorganization and recruitment in different ways (Steward et al., 2011). Obviously, one type of mechanical load alone is unlikely sufficient to generate the required mechanotransductive signaling events for a specific function phenotype. An example to echo this point is the combinatory regulations of surface shear (±25° oscillation at 1 Hz) and cyclic axial compression (0.4 mm amplitude or 10%–20% strain at 1 Hz) for chondrogenic differentiation of hBMSCs. Here the combination enables to mediate chondrogenic gene expression and sulphated glycosaminoglycan and Col-II deposit but either alone does not work (Schatti et al., 2011). Moreover, chondrosarcoma cells yield a less profound effect on the gene expression profile under hydrostatic pressure (continuous and 0.5 Hz cyclic pressure at 5 MPa) than that under cyclic stretch (8% strain at 0.5 Hz) (Karjalainen et al., 2003).

**Figure 2:**
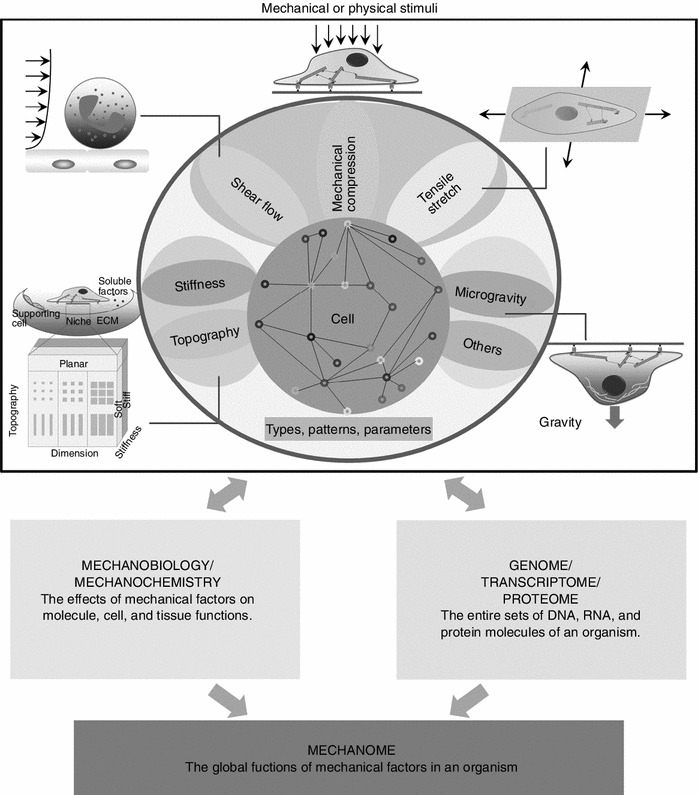
**Conceptual demonstration of mechanome to illustrate the combination of mechanobiology/mechanochemistry and genome/transcriptome/proteome**. Different mechanical stimuli mediate distinct responsive functions at molecule, cell, or tissue level, while the omics techniques map the entire sets of molecular events of an organism. The combined field helps to uncover globally the mysteries of mechanobiology and mechanochemistry from the viewpoint of omics analyses at different levels

To date, cell mechanotransduction has attracted much attentions to understand, from both theoretical and experimental aspects, the mechanisms how a cell adapts the mechanical stimuli and how the mechanical microenvironments are remodeled. The progress in combining mechanobiology with omics approaches also demonstrates that omics analysis is efficient in screening mechanosensitive genes or proteins in various mechanical stimuli (Fig. 2).

## Progresses and Perspective of Mechanomics

Omics approaches, such as transcriptomics and proteomics, are powerful system-level tools that can be applied not only in biology and medicine but also in mechanobiology. However, no system-level omics definition is proposed to summarize the roles of mechanical forces in biological processes, i.e., mechanome. Mapping the mechanome of cells furthers the understandings of mechanosensation and mechanotransduction. Brief discussion is conducted here since only few papers are currently published on this special topic.

### Concept of mechanomics

The definition of mechanome is originated for globally elucidating the functions of mechanical forces existing *in vivo* at cellular and molecular scales (Lang, 2007; Song et al., 2012, 2013). It is then extended to describe the events in tissue, organ, or even whole organism, aiming to understand ultimately the roles of mechanical stress from nano- and micro- to macro-scopic viewpoints (Song et al., 2012). It is necessary to collect the information of mechanical force distribution at different levels, and map the cross-talks between mechanical forces and biological functions. This turns out to a new field, mechanomics. The term mechanomics was first proposed for combining the nuclear magnetic resonance technique with bioinformatics strategies to characterize the protein-ligand interactions across large families of proteins (Sem et al., 2001). Evidently, such definition is distinct with what we discuss here. Regardless of this, the term can be borrowed for describing the mechanically-induced events at multiple levels. Fig. 2 demonstrates the conceptual flowchart of this emerging field. It seeks to understand the fundamental mechanical or physical processes that are common to biological function and to study how forces are transmitted and transduced to regulate biological responses. A similar term of physicomics was also proposed to deal with physical parameters such as pressure, temperature, electromagnetic fields etc. that are involved in cell, tissue, or physiology (van Loon, 2009). To date, quite a few works from the viewpoint of mechanomics are found on transcriptomic and proteomic analyses for the combined mechanical stimuli. Mapping the global responses is critical since multiple mechanical stimuli are associated with the multiple cellular and molecular events (Fig. 2).

### Limited progresses in the fields related to mechanomics

Since the term mechanomics is proposed from a distinct viewpoint of drug design (Sem et al., 2001), it has been submerged away from the community of mechanobiology for a while. To date, only a few works are reported along the line described here. One example is that understanding the roles of mechanical forces and machinery (e.g., biological motors and polymerization of filament) opens the window to new strategies for molecular medicine (Wang et al., 2008; Huang et al., 2013). Two more examples are to map the mechanome of live stem cells using both fluorescent microbeads and computational fluid dynamics (CFD) simulation and to measure local strain fields *in situ* at the fluid-cell interface, which provides mechanistic insights into the roles of mechanical forces in lineage commitment as it unfolds (Wang et al., 2008; Song et al., 2012, 2013). In fact, a body of works, such as those aforementioned, have already adopted the idea or concept of mechanomics to elucidate the cellular and molecular events on different mechanical stimuli even though the term mechanomics is not used explicitly.

### Biomechanical and biological approaches appropriate for mechanomics

Biomechanical assays and techniques developed previously are able to be applied in mechanomics. Shear flow, tensile stretch, and mechanical compression are most frequently tested using in-house developed or commercial instruments such as parallel flow chamber, membrane-stretch apparatus, or osmotic/hydrostatic compression device. As indicated in the literatures (van Loon, 2009; Long et al., 2011), parallel or disk flow chamber is used to mimic physiological blood or interstitial flow, uni-, bi-, or equi-axial stretch apparatus is applied to initiate the substrate or ECM tension, and tubular or spheroidal compression device is employed to represent physiological compression. In addition to these approaches, micropipette suction assay applies forces on the cell or vacuole membrane by deforming them (Huang et al., 2004; Fu et al., 2011b). Optical tweezers utilize an optical gradient to trap and exert forces on refractive microbeads (Zhang et al., 2008; Sun et al., 2009; Li et al., 2013b) and magnetic tweezers apply twisting forces *via* a magnetic field to firmly-attached magnetic microbeads on cell surface (Grossi et al., 2011). Atomic force microscopy enables to quantify rupture force/bond lifetime of protein-protein interactions at single molecule level (Lü et al., 2006). Besides, microgravity simulators such as clinostat, rotating-wall bioreactor, random positioning machine, or magnetic levitation are considered to stimulate the space microgravity effects for ground-based species while hypergravity centrifuges are useful tools for mimicking the hypergravity impacts in space life sciences. Micro-patterned or micro-fabricated substrates are assumed to mimic the physiological topography of microenvironment or niche and then applied in mechanobiology studies of cell proliferation and differentiation (Li et al., 2013c). Meanwhile, coupling the typical fluorescent assays such as fluorescence resonance energy transfer with those mechanical assays also enables to visualize the intracellular *in situ* events under given mechanical stimuli.

Transcriptomic/proteomic approaches are also critical for the study of mechanomics. Much progress has been made from routine chip-based analysis to the advanced RNA-seq techniques (e.g., SAGE) or from conventional 2-D gel assay to the hi-tech proteomic techniques (e.g., iTRAQ, and LC-MS/MS).

### Future perspectives of mechanomics

Mechanomics is a young but fast developing field. Promising clues are emerging in its related aspects in recent years. The framework is exemplified in Fig. 2 to clarify how all these related fields can be linked together. While there should be lots of works to pursue, several potential issues are proposed from the above discussions.

Biological or physiological significance yield the top priority in mechanomic studies. It is little known about how mechanical forces appearing in cell or tissue contribute to development, physiology, and diseases. Molecular mechanisms (e.g., mechanosensors) by which cells sense and respond to mechanical signals is the major challenge we are facing, since we are still far away to establish the responsive network under global mechanical stimuli. More biological models should be considered, as the biologists usually do, to broaden the vision of study for biological diversity. Expression profiling under pathological mechanical conditions is another hotspot in the mechanobiology of tumor growth (Carey et al., 2012).

Replication of the *in vivo* mechanical patterns or parameters is also a prior challenge to confine the understanding how mechanomics works for cells. In a living organism, the *in vivo* mechanical environment is quite complicated and usually coupled with other physical and chemical factors. Thus, it is difficult to replicate the local biomechanical environment that surrounds cells or comprises tissues and is hard to determine the physiological mechanical patterns or parameters. Meanwhile, existing *in vitro* data for cells exposed to mechanical stimuli are usually compared with those under static culture conditions, which require the careful design of control cases that are expected to subtract reasonably background noises from measured signals. More attentions should also be paid for those stimuli not indicated here (e.g., vibration, sound, touch).

Developing new *in vitro* assays specific for mechanomics is also an important issue. New hi-tech techniques in related fields provide the opportunities for promoting the mechanomics. On-chip strain sensors developed by micro-fabrication combined with mechanical tension promise to test small samples and mechanical loading in parallel, to reduce the labor-consumption, and to enhance testing efficiency (MacQueen et al., 2012). Micro-fabricated composite material screening array is also able to determine the combined effects of substrate stretch, soluble cues, and matrix proteins on small populations of primary cells, which eventually enable us to profile gene expression from a single cell (Moraes et al., 2013). Meanwhile, the state-of-art techniques in biology direct the analyses of cells *in situ* or *in vivo,* or carry out immediately the post mortem to retain the features of the native environment. It is then possible to design appropriate *in vitro* experiments that critically test the most dominant mechanical characteristics existing *in vivo.* New hi-tech techniques of 2nd-generation sequencing or single-cell sequencing could be efficient candidates for mapping the mechanomics of various species (Lawrie et al., 2008).

Large-scale data mining and bioinformatics analysis are indispensable in this field. To facilitate the integration of database, an open-access and downloadable mechanomics database should be created worldwide, serving as a web-based resource like NCBI that collates quantitative analyses of mechanomics. One key feature of the database is able to map globally the mechanical environment of cells and provide the quantitative information for transcripts and proteins from various sources. Another is the capability of large-scale, elaborated analyses with interactive interface for the users who attempt to mine the existing data or submit newly measured data using various search engines or experimental assays.

## Conclusions

Combination of mechanobiology and transcriptomics/proteomics is an emerging field for globally understanding the gene expression and protein production in response to multiple types of mechanical stimuli. Evidently, mechanomics is still in its embryonic stage. With the high-throughput analyses in transcriptomics and proteomics and the state-of-art techniques in biomechanics, one expects to define the required mechanical variables and to provide an integrated profile of signaling events from the viewpoint of mechanome at molecular and cellular levels.

## Abbreviations

2-D: two dimensional
ACL: anterior cruciate ligament
EC: endothelial cell
ECM: extracellular matrix
MPC: mesenchymal progenitor cell
HSP: heat shock protein
IGF-I: insulin-like growth factor I
iTRAQ: isobaric tags for relative and absolute quantification
LC: liquid chromatograph
MS: mass spectrometry
MSC: mesenchymal stem cell
SAGE: serial analysis of gene expression
SMC: smooth muscle cell
